# Supplementary material for: Mapping the evolution of fertility support policies in China: A content and instrumental analysis
Source: PLoS One. 2025 Oct 9;20(10):e0332137. doi: 10.1371/journal.pone.0332137 (PMC12510515; doi:10.1371/journal.pone.0332137)
Supplement: S1 Appendix — (ZIP) [file pone.0332137.s001.zip › S1 Appendix. 226 original policy documents/156-中华人民共和国劳动法(2009修正)(FBM-CLI-1-167200).doc]

中华人民共和国劳动法(2009修正)

**发布部门：** [全国人大常委会](https://www.pkulaw.com/law/chl?Aggs.IssueDepartment=201)

**发文字号：**中华人民共和国主席令第18号

**发布日期：**2009.08.27

**实施日期：**2009.08.27

**时效性：** [已被修改](https://www.pkulaw.com/law/chl?Aggs.TimelinessDic=03)

**效力级别：** [法律](https://www.pkulaw.com/law/chl?Aggs.EffectivenessDic=XA0101)

**法规类别：** [劳动工会综合规定](https://www.pkulaw.com/law/chl?Aggs.Category=08701) [营商环境优化](https://dobusiness.pkulaw.com/)

**修改依据：**[全国人民代表大会常务委员会关于修改《中华人民共和国劳动法》等七部法律的决定(含中华人民共和国老年人权益保障法、中华人民共和国环境噪声污染防治法、中华人民共和国环境影响评价法、中华人民共和国民办教育促进法、中华人民共和国民用航空法、中华人民共和国职业病防治法)](javascript:TMP(328151,0,'chl'))

**法宝提示：**本篇法规的变更情况请参考：[国务院关于第三批取消中央指定地方实施行政许可事项的决定](javascript:TMP(289223,0,'chl'))

[2009-2018编注](https://www.pkulaw.com/history/compare/编注-241.html) [1994-2009编注](https://www.pkulaw.com/history/compare/编注-240.html)

中华人民共和国劳动法
(1994年7月5日第八届全国人民代表大会常务委员会第八次会议通过 1994年7月5日中华人民共和国主席令第二十八号公布 根据2009年8月27日中华人民共和国主席令第十八号第十一届全国人民代表大会常务委员会第十次会议《关于修改部分法律的决定》修正)

目录

　　第一章　总则
　　第二章　促进就业
　　第三章　劳动合同和集体合同
　　第四章　工作时间和休息休假
　　第五章　工资
　　第六章　劳动安全卫生
　　第七章　女职工和未成年工特殊保护
　　第八章　职业培训
　　第九章　社会保险和福利
　　第十章　劳动争议
　　第十一章　监督检查
　　第十二章　法律责任
　　第十三章　附则

第一章　总则

　　**第一条**　为了保护劳动者的合法权益，调整劳动关系，建立和维护适应社会主义市场经济的劳动制度，促进经济发展和社会进步，根据宪法，制定本法。

　　**第二条**　在中华人民共和国境内的企业、个体经济组织（以下统称用人单位）和与之形成劳动关系的劳动者，适用本法。

　　国家机关、事业组织、社会团体和与之建立劳动合同关系的劳动者，依照本法执行。

　　**第三条**　劳动者享有平等就业和选择职业的权利、取得劳动报酬的权利、休息休假的权利、获得劳动安全卫生保护的权利、接受职业技能培训的权利、享受社会保险和福利的权利、提请劳动争议处理的权利以及法律规定的其他劳动权利。

　　劳动者应当完成劳动任务，提高职业技能，执行劳动安全卫生规程，遵守劳动纪律和职业道德。

　　**第四条**　用人单位应当依法建立和完善规章制度，保障劳动者享有劳动权利和履行劳动义务。

　　**第五条**　国家采取各种措施，促进劳动就业，发展职业教育，制定劳动标准，调节社会收入，完善社会保险，协调劳动关系，逐步提高劳动者的生活水平。

　　**第六条**　国家提倡劳动者参加社会义务劳动，开展劳动竞赛和合理化建议活动，鼓励和保护劳动者进行科学研究、技术革新和发明创造，表彰和奖励劳动模范和先进工作者。

　　**第七条**　劳动者有权依法参加和组织工会。

　　工会代表和维护劳动者的合法权益，依法独立自主地开展活动。

　　**第八条**　劳动者依照法律规定，通过职工大会、职工代表大会或者其他形式，参与民主管理或者就保护劳动者合法权益与用人单位进行平等协商。

　　**第九条**　国务院劳动行政部门主管全国劳动工作。

　　县级以上地方人民政府劳动行政部门主管本行政区域内的劳动工作。

第二章　促进就业

　　**第十条**　国家通过促进经济和社会发展，创造就业条件，扩大就业机会。

　　国家鼓励企业、事业组织、社会团体在法律、行政法规规定的范围内兴办产业或者拓展经营，增加就业。

　　国家支持劳动者自愿组织起来就业和从事个体经营实现就业。

　　**第十一条**　地方各级人民政府应当采取措施，发展多种类型的职业介绍机构，提供就业服务。

　　**第十二条**　劳动者就业，不因民族、种族、性别、宗教信仰不同而受歧视。

　　**第十三条**　妇女享有与男子平等的就业权利。在录用职工时，除国家规定的不适合妇女的工种或者岗位外，不得以性别为由拒绝录用妇女或者提高对妇女的录用标准。

　　**第十四条**　残疾人、少数民族人员、退出现役的军人的就业，法律、法规有特别规定的，从其规定。

　　**第十五条**　禁止用人单位招用未满十六周岁的未成年人。

　　文艺、体育和特种工艺单位招用未满十六周岁的未成年人，必须依照国家有关规定，履行审批手续，并保障其接受义务教育的权利。

第三章　劳动合同和集体合同

　　**第十六条**　劳动合同是劳动者与用人单位确立劳动关系、明确双方权利和义务的协议。

　　建立劳动关系应当订立劳动合同。

　　**第十七条**　订立和变更劳动合同，应当遵循平等自愿、协商一致的原则，不得违反法律、行政法规的规定。

　　劳动合同依法订立即具有法律约束力，当事人必须履行劳动合同规定的义务。

　　**第十八条**　下列劳动合同无效：

　　（一）违反法律、行政法规的劳动合同；

　　（二）采取欺诈、威胁等手段订立的劳动合同。

　　无效的劳动合同，从订立的时候起，就没有法律约束力。确认劳动合同部分无效的，如果不影响其余部分的效力，其余部分仍然有效。

　　劳动合同的无效，由劳动争议仲裁委员会或者人民法院确认。

　　**第十九条**　劳动合同应当以书面形式订立，并具备以下条款：

　　（一）劳动合同期限；

　　（二）工作内容；

　　（三）劳动保护和劳动条件；

　　（四）劳动报酬；

　　（五）劳动纪律；

　　（六）劳动合同终止的条件；

　　（七）违反劳动合同的责任。

　　劳动合同除前款规定的必备条款外，当事人可以协商约定其他内容。

　　**第二十条**　劳动合同的期限分为有固定期限、无固定期限和以完成一定的工作为期限。

　　劳动者在同一用人单位连续工作满十年以上，当事人双方同意续延劳动合同的，如果劳动者提出订立无固定期限的劳动合同，应当订立无固定期限的劳动合同。

　　**第二十一条**　劳动合同可以约定试用期。试用期最长不得超过六个月。

　　**第二十二条**　劳动合同当事人可以在劳动合同中约定保守用人单位商业秘密的有关事项。

　　**第二十三条**　劳动合同期满或者当事人约定的劳动合同终止条件出现，劳动合同即行终止。

　　**第二十四条**　经劳动合同当事人协商一致，劳动合同可以解除。

　　**第二十五条**　劳动者有下列情形之一的，用人单位可以解除劳动合同：

　　（一）在试用期间被证明不符合录用条件的；

　　（二）严重违反劳动纪律或者用人单位规章制度的；

　　（三）严重失职，营私舞弊，对用人单位利益造成重大损害的；

　　（四）被依法追究刑事责任的。

　　**第二十六条**　有下列情形之一的，用人单位可以解除劳动合同，但是应当提前三十日以书面形式通知劳动者本人：

　　（一）劳动者患病或者非因工负伤，医疗期满后，不能从事原工作也不能从事由用人单位另行安排的工作的；

　　（二）劳动者不能胜任工作，经过培训或者调整工作岗位，仍不能胜任工作的；

　　（三）劳动合同订立时所依据的客观情况发生重大变化，致使原劳动合同无法履行，经当事人协商不能就变更劳动合同达成协议的。

　　**第二十七条**　用人单位濒临破产进行法定整顿期间或者生产经营状况发生严重困难，确需裁减人员的，应当提前三十日向工会或者全体职工说明情况，听取工会或者职工的意见，经向劳动行政部门报告后，可以裁减人员。

　　用人单位依据本条规定裁减人员，在六个月内录用人员的，应当优先录用被裁减的人员。

　　**第二十八条**　用人单位依据本法第二十四条、第二十六条、第二十七条的规定解除劳动合同的，应当依照国家有关规定给予经济补偿。

　　**第二十九条**　劳动者有下列情形之一的，用人单位不得依据本法第二十六条、第二十七条的规定解除劳动合同：

　　（一）患职业病或者因工负伤并被确认丧失或者部分丧失劳动能力的；

　　（二）患病或者负伤，在规定的医疗期内的；

　　（三）女职工在孕期、产期、哺乳期内的；

　　（四）法律、行政法规规定的其他情形。

　　**第三十条**　用人单位解除劳动合同，工会认为不适当的，有权提出意见。如果用人单位违反法律、法规或者劳动合同，工会有权要求重新处理；劳动者申请仲裁或者提起诉讼的，工会应当依法给予支持和帮助。

　　**第三十一条**　劳动者解除劳动合同，应当提前三十日以书面形式通知用人单位。

　　**第三十二条**　有下列情形之一的，劳动者可以随时通知用人单位解除劳动合同：

　　（一）在试用期内的；

　　（二）用人单位以暴力、威胁或者非法限制人身自由的手段强迫劳动的；

　　（三）用人单位未按照劳动合同约定支付劳动报酬或者提供劳动条件的。

　　**第三十三条**　企业职工一方与企业可以就劳动报酬、工作时间、休息休假、劳动安全卫生、保险福利等事项，签订集体合同。集体合同草案应当提交职工代表大会或者全体职工讨论通过。

　　集体合同由工会代表职工与企业签订；没有建立工会的企业，由职工推举的代表与企业签订。

　　**第三十四条**　集体合同签订后应当报送劳动行政部门；劳动行政部门自收到集体合同文本之日起十五日内未提出异议的，集体合同即行生效。

　　**第三十五条**　依法签订的集体合同对企业和企业全体职工具有约束力。职工个人与企业订立的劳动合同中劳动条件和劳动报酬等标准不得低于集体合同的规定。

第四章　工作时间和休息休假

　　**第三十六条**　国家实行劳动者每日工作时间不超过八小时、平均每周工作时间不超过四十四小时的工时制度。

　　**第三十七条**　对实行计件工作的劳动者，用人单位应当根据本法第三十六条规定的工时制度合理确定其劳动定额和计件报酬标准。

　　**第三十八条**　用人单位应当保证劳动者每周至少休息一日。

　　**第三十九条**　企业因生产特点不能实行本法第三十六条、第三十八条规定的，经劳动行政部门批准，可以实行其他工作和休息办法。

　　**第四十条**　用人单位在下列节日期间应当依法安排劳动者休假：

　　（一）元旦；

　　（二）春节；

　　（三）国际劳动节；

　　（四）国庆节；

　　（五）法律、法规规定的其他休假节日。

　　**第四十一条**　用人单位由于生产经营需要，经与工会和劳动者协商后可以延长工作时间，一般每日不得超过一小时；因特殊原因需要延长工作时间的，在保障劳动者身体健康的条件下延长工作时间每日不得超过三小时，但是每月不得超过三十六小时。

　　**第四十二条**　有下列情形之一的，延长工作时间不受本法第四十一条规定的限制：

　　（一）发生自然灾害、事故或者因其他原因，威胁劳动者生命健康和财产安全，需要紧急处理的；

　　（二）生产设备、交通运输线路、公共设施发生故障，影响生产和公众利益，必须及时抢修的；

　　（三）法律、行政法规规定的其他情形。

　　**第四十三条**　用人单位不得违反本法规定延长劳动者的工作时间。

　　**第四十四条**　有下列情形之一的，用人单位应当按照下列标准支付高于劳动者正常工作时间工资的工资报酬：

　　（一）安排劳动者延长工作时间的，支付不低于工资的百分之一百五十的工资报酬；

　　（二）休息日安排劳动者工作又不能安排补休的，支付不低于工资的百分之二百的工资报酬；

　　（三）法定休假日安排劳动者工作的，支付不低于工资的百分之三百的工资报酬。

　　**第四十五条**　国家实行带薪年休假制度。劳动者连续工作一年以上的，享受带薪年休假。具体办法由国务院规定。

第五章　工资

　　**第四十六条**　工资分配应当遵循按劳分配原则，实行同工同酬。

　　工资水平在经济发展的基础上逐步提高。国家对工资总量实行宏观调控。

　　**第四十七条**　用人单位根据本单位的生产经营特点和经济效益，依法自主确定本单位的工资分配方式和工资水平。

　　**第四十八条**　国家实行最低工资保障制度。最低工资的具体标准由省、自治区、直辖市人民政府规定，报国务院备案。

　　用人单位支付劳动者的工资不得低于当地最低工资标准。

　　**第四十九条**　确定和调整最低工资标准应当综合参考下列因素：

　　（一）劳动者本人及平均赡养人口的最低生活费用；

　　（二）社会平均工资水平；

　　（三）劳动生产率；

　　（四）就业状况；

　　（五）地区之间经济发展水平的差异。

　　**第五十条**　工资应当以货币形式按月支付给劳动者本人。不得克扣或者无故拖欠劳动者的工资。

　　**第五十一条**　劳动者在法定休假日和婚丧假期间以及依法参加社会活动期间，用人单位应当依法支付工资。

第六章　劳动安全卫生

　　**第五十二条**　用人单位必须建立、健全劳动安全卫生制度，严格执行国家劳动安全卫生规程和标准，对劳动者进行劳动安全卫生教育，防止劳动过程中的事故，减少职业危害。

　　**第五十三条**　劳动安全卫生设施必须符合国家规定的标准。

　　新建、改建、扩建工程的劳动安全卫生设施必须与主体工程同时设计、同时施工、同时投入生产和使用。

　　**第五十四条**　用人单位必须为劳动者提供符合国家规定的劳动安全卫生条件和必要的劳动防护用品，对从事有职业危害作业的劳动者应当定期进行健康检查。

　　**第五十五条**　从事特种作业的劳动者必须经过专门培训并取得特种作业资格。

　　**第五十六条**　劳动者在劳动过程中必须严格遵守安全操作规程。

　　劳动者对用人单位管理人员违章指挥、强令冒险作业，有权拒绝执行；对危害生命安全和身体健康的行为，有权提出批评、检举和控告。

　　**第五十七条**　国家建立伤亡事故和职业病统计报告和处理制度。县级以上各级人民政府劳动行政部门、有关部门和用人单位应当依法对劳动者在劳动过程中发生的伤亡事故和劳动者的职业病状况，进行统计、报告和处理。

第七章　女职工和未成年工特殊保护

　　**第五十八条**　国家对女职工和未成年工实行特殊劳动保护。

　　未成年工是指年满十六周岁未满十八周岁的劳动者。

　　**第五十九条**　禁止安排女职工从事矿山井下、国家规定的第四级体力劳动强度的劳动和其他禁忌从事的劳动。

　　**第六十条**　不得安排女职工在经期从事高处、低温、冷水作业和国家规定的第三级体力劳动强度的劳动。

　　**第六十一条**　不得安排女职工在怀孕期间从事国家规定的第三级体力劳动强度的劳动和孕期禁忌从事的劳动。对怀孕七个月以上的女职工，不得安排其延长工作时间和夜班劳动。

　　**第六十二条**　女职工生育享受不少于九十天的产假。

　　**第六十三条**　不得安排女职工在哺乳未满一周岁的婴儿期间从事国家规定的第三级体力劳动强度的劳动和哺乳期禁忌从事的其他劳动，不得安排其延长工作时间和夜班劳动。

　　**第六十四条**　不得安排未成年工从事矿山井下、有毒有害、国家规定的第四级体力劳动强度的劳动和其他禁忌从事的劳动。

　　**第六十五条**　用人单位应当对未成年工定期进行健康检查。

第八章　职业培训

　　**第六十六条**　国家通过各种途径，采取各种措施，发展职业培训事业，开发劳动者的职业技能，提高劳动者素质，增强劳动者的就业能力和工作能力。

　　**第六十七条**　各级人民政府应当把发展职业培训纳入社会经济发展的规划，鼓励和支持有条件的企业、事业组织、社会团体和个人进行各种形式的职业培训。

　　**第六十八条**　用人单位应当建立职业培训制度，按照国家规定提取和使用职业培训经费，根据本单位实际，有计划地对劳动者进行职业培训。

　　从事技术工种的劳动者，上岗前必须经过培训。

　　**第六十九条**　国家确定职业分类，对规定的职业制定职业技能标准，实行职业资格证书制度，由经过政府批准的考核鉴定机构负责对劳动者实施职业技能考核鉴定。

第九章　社会保险和福利

　　**第七十条**　国家发展社会保险事业，建立社会保险制度，设立社会保险基金，使劳动者在年老、患病、工伤、失业、生育等情况下获得帮助和补偿。

　　**第七十一条**　社会保险水平应当与社会经济发展水平和社会承受能力相适应。

　　**第七十二条**　社会保险基金按照保险类型确定资金来源，逐步实行社会统筹。用人单位和劳动者必须依法参加社会保险，缴纳社会保险费。

　　**第七十三条**　劳动者在下列情形下，依法享受社会保险待遇：

　　（一）退休；

　　（二）患病、负伤；

　　（三）因工伤残或者患职业病；

　　（四）失业；

　　（五）生育。

　　劳动者死亡后，其遗属依法享受遗属津贴。

　　劳动者享受社会保险待遇的条件和标准由法律、法规规定。

　　劳动者享受的社会保险金必须按时足额支付。

　　**第七十四条**　社会保险基金经办机构依照法律规定收支、管理和运营社会保险基金，并负有使社会保险基金保值增值的责任。

　　社会保险基金监督机构依照法律规定，对社会保险基金的收支、管理和运营实施监督。

　　社会保险基金经办机构和社会保险基金监督机构的设立和职能由法律规定。

　　任何组织和个人不得挪用社会保险基金。

　　**第七十五条**　国家鼓励用人单位根据本单位实际情况为劳动者建立补充保险。

　　国家提倡劳动者个人进行储蓄性保险。

　　**第七十六条**　国家发展社会福利事业，兴建公共福利设施，为劳动者休息、休养和疗养提供条件。

　　用人单位应当创造条件，改善集体福利，提高劳动者的福利待遇。

第十章　劳动争议

　　**第七十七条**　用人单位与劳动者发生劳动争议，当事人可以依法申请调解、仲裁、提起诉讼，也可以协商解决。

　　调解原则适用于仲裁和诉讼程序。

　　**第七十八条**　解决劳动争议，应当根据合法、公正、及时处理的原则，依法维护劳动争议当事人的合法权益。

　　**第七十九条**　劳动争议发生后，当事人可以向本单位劳动争议调解委员会申请调解；调解不成，当事人一方要求仲裁的，可以向劳动争议仲裁委员会申请仲裁。当事人一方也可以直接向劳动争议仲裁委员会申请仲裁。对仲裁裁决不服的，可以向人民法院提起诉讼。

　　**第八十条**　在用人单位内，可以设立劳动争议调解委员会。劳动争议调解委员会由职工代表、用人单位代表和工会代表组成。劳动争议调解委员会主任由工会代表担任。

　　劳动争议经调解达成协议的，当事人应当履行。

　　**第八十一条**　劳动争议仲裁委员会由劳动行政部门代表、同级工会代表、用人单位方面的代表组成。劳动争议仲裁委员会主任由劳动行政部门代表担任。

　　**第八十二条**　提出仲裁要求的一方应当自劳动争议发生之日起六十日内向劳动争议仲裁委员会提出书面申请。仲裁裁决一般应在收到仲裁申请的六十日内作出。对仲裁裁决无异议的，当事人必须履行。

　　**第八十三条**　劳动争议当事人对仲裁裁决不服的，可以自收到仲裁裁决书之日起十五日内向人民法院提起诉讼。一方当事人在法定期限内不起诉又不履行仲裁裁决的，另一方当事人可以申请人民法院强制执行。

　　**第八十四条**　因签订集体合同发生争议，当事人协商解决不成的，当地人民政府劳动行政部门可以组织有关各方协调处理。

　　因履行集体合同发生争议，当事人协商解决不成的，可以向劳动争议仲裁委员会申请仲裁；对仲裁裁决不服的，可以自收到仲裁裁决书之日起十五日内向人民法院提起诉讼。

第十一章　监督检查

　　**第八十五条**　县级以上各级人民政府劳动行政部门依法对用人单位遵守劳动法律、法规的情况进行监督检查，对违反劳动法律、法规的行为有权制止，并责令改正。

　　**第八十六条**　县级以上各级人民政府劳动行政部门监督检查人员执行公务，有权进入用人单位了解执行劳动法律、法规的情况，查阅必要的资料，并对劳动场所进行检查。

　　县级以上各级人民政府劳动行政部门监督检查人员执行公务，必须出示证件，秉公执法并遵守有关规定。

　　**第八十七条**　县级以上各级人民政府有关部门在各自职责范围内，对用人单位遵守劳动法律、法规的情况进行监督。

　　**第八十八条**　各级工会依法维护劳动者的合法权益，对用人单位遵守劳动法律、法规的情况进行监督。

　　任何组织和个人对于违反劳动法律、法规的行为有权检举和控告。

第十二章　法律责任

　　**第八十九条**　用人单位制定的劳动规章制度违反法律、法规规定的，由劳动行政部门给予警告，责令改正；对劳动者造成损害的，应当承担赔偿责任。

　　**第九十条**　用人单位违反本法规定，延长劳动者工作时间的，由劳动行政部门给予警告，责令改正，并可以处以罚款。

　　**第九十一条**　用人单位有下列侵害劳动者合法权益情形之一的，由劳动行政部门责令支付劳动者的工资报酬、经济补偿，并可以责令支付赔偿金：

　　（一）克扣或者无故拖欠劳动者工资的；

　　（二）拒不支付劳动者延长工作时间工资报酬的；

　　（三）低于当地最低工资标准支付劳动者工资的；

　　（四）解除劳动合同后，未依照本法规定给予劳动者经济补偿的。

　　**第九十二条**　用人单位的劳动安全设施和劳动卫生条件不符合国家规定或者未向劳动者提供必要的劳动防护用品和劳动保护设施的，由劳动行政部门或者有关部门责令改正，可以处以罚款；情节严重的，提请县级以上人民政府决定责令停产整顿；对事故隐患不采取措施，致使发生重大事故，造成劳动者生命和财产损失的，对责任人员依照刑法有关规定追究刑事责任。

　　**第九十三条**　用人单位强令劳动者违章冒险作业，发生重大伤亡事故，造成严重后果的，对责任人员依法追究刑事责任。

　　**第九十四条**　用人单位非法招用未满十六周岁的未成年人的，由劳动行政部门责令改正，处以罚款；情节严重的，由工商行政管理部门吊销营业执照。

　　**第九十五条**　用人单位违反本法对女职工和未成年工的保护规定，侵害其合法权益的，由劳动行政部门责令改正，处以罚款；对女职工或者未成年工造成损害的，应当承担赔偿责任。

　　**第九十六条**　用人单位有下列行为之一，由公安机关对责任人员处以十五日以下拘留、罚款或者警告；构成犯罪的，对责任人员依法追究刑事责任：

　　（一）以暴力、威胁或者非法限制人身自由的手段强迫劳动的；

　　（二）侮辱、体罚、殴打、非法搜查和拘禁劳动者的。

　　**第九十七条**　由于用人单位的原因订立的无效合同，对劳动者造成损害的，应当承担赔偿责任。

　　**第九十八条**　用人单位违反本法规定的条件解除劳动合同或者故意拖延不订立劳动合同的，由劳动行政部门责令改正；对劳动者造成损害的，应当承担赔偿责任。

　　**第九十九条**　用人单位招用尚未解除劳动合同的劳动者，对原用人单位造成经济损失的，该用人单位应当依法承担连带赔偿责任。

　　**第一百条**　用人单位无故不缴纳社会保险费的，由劳动行政部门责令其限期缴纳，逾期不缴的，可以加收滞纳金。

　　**第一百零一条**　用人单位无理阻挠劳动行政部门、有关部门及其工作人员行使监督检查权，打击报复举报人员的，由劳动行政部门或者有关部门处以罚款；构成犯罪的，对责任人员依法追究刑事责任。

　　**第一百零二条**　劳动者违反本法规定的条件解除劳动合同或者违反劳动合同中约定的保密事项，对用人单位造成经济损失的，应当依法承担赔偿责任。

　　**第一百零三条**　劳动行政部门或者有关部门的工作人员滥用职权、玩忽职守、徇私舞弊，构成犯罪的，依法追究刑事责任；不构成犯罪的，给予行政处分。

　　**第一百零四条**　国家工作人员和社会保险基金经办机构的工作人员挪用社会保险基金，构成犯罪的，依法追究刑事责任。

　　**第一百零五条**　违反本法规定侵害劳动者合法权益，其他法律、行政法规已规定处罚的，依照该法律、行政法规的规定处罚。

第十三章　附则

　　**第一百零六条**　省、自治区、直辖市人民政府根据本法和本地区的实际情况，规定劳动合同制度的实施步骤，报国务院备案。

　　**第一百零七条**　本法自1995年1月1日起施行。

© 北大法宝：（[www.pkulaw.com](https://www.pkulaw.com/)) 专业提供法律信息、法学知识和法律软件领域各类解决方案。北大法宝为您提供丰富的参考资料，正式引用法规条文时请与标准文本核对。
欢迎查看所有[产品和服务](https://www.pkulaw.com/helps/2.html)。法宝快讯：[如何快速找到您需要的检索结果？](https://www.pkulaw.com/helps/70.html)[法宝](https://www.pkulaw.com/helps/69.html)V6有何新特色？

扫描二维码阅读原文

**原文链接：**<https://www.pkulaw.com/chl/b512be6605f576c3bdfb.html>
